# Assessment of variable drinking water sources used in Egypt on broiler health and welfare

**DOI:** 10.14202/vetworld.2015.855-864

**Published:** 2015-07-14

**Authors:** N. ELSaidy, R. A. Mohamed, F. Abouelenien

**Affiliations:** 1 Department of Hygiene and Preventive Medicine, Faculty of Veterinary Medicine, Kafrelsheikh University, Kafr El-Sheikh Governorate - 33156, Egypt; 2 Department of Hygiene and Preventive Medicine (Animal Behavior and Welfare), Faculty of Veterinary Medicine, Kafrelsheikh University, Kafr El-sheikh City - 33156, Egypt

**Keywords:** broiler performance, broiler health and immunity, poultry drinking water, water treatment

## Abstract

**Aim:**

This study assessed the impact of four water sources used as drinking water in Egypt for broiler chickens on its performance, carcass characteristic, hematological, and immunological responses.

**Materials and Methods:**

A total of 204 unsexed 1-day old Indian River broiler chickens were used in this study. They were randomly allocated into four treatment groups of 51 birds in each, with three replicates, 17 birds per replicate. Groups were classified according to water source they had been received into (T1) received farm tap water; (T2) received filtered tap water (T3) received farm stored water at rooftop tanks, (T4) received underground (well) water.

**Results:**

All water sources showed no significant differences among treated groups at (p>0.05) for most of the performance parameters and carcass characteristics. However (T2) group showed higher records for body weight (BWT), BWT gain (BWG), feed conversion ratio, bursa weight, serum total protein, globulin (G), albumin (A) and A/G ratio, Ab titer against New castle disease virus vaccine. On the other hand, it showed lower records for water intake (WI), WI/Feed intake ratio, total leukocytes count %, heterophil %, lymphocyte %, H/L ratio, liver weight, glutamic oxaloacetic transaminase, glutamic pyruvic transaminase, serum uric acid and creatinine. Where filtered water reverse osmosis showed lowest records for bacterial load, the absence of coliform bacteria, total dissolved solids (TDS), electrical conductivity (EC) and salinity. On the other hand stored water showed higher numerical values for TDS, EC, alkalinity, salinity, pH, bacterial count, and coliform count.

**Conclusion:**

Base on the results of this study, it is concluded that different water sources could safely be used as drinking water for poultry; as long as it is present within the acceptable range of drinking water quality for chickens. Suggesting the benefits of treatment of water sources on improving chickens’ health and welfare. Draw attention to the importance of maintaining the hygienic quality of stored water.

## Introduction

Water is the most critical and vital nutrient to health and wellbeing [1]. Water is involved in many aspects of poultry metabolism including body temperature control, food digestion and absorption, nutrients transport, and waste products elimination from the body [2]. Birds consume approximately 1.6-2.0 times as much water as feed on a weight basis [3]; therefore, any deviation in water quality could have a more pronounced effect on poultry health and production than feed did. Drinking water is of concern to poultry producers due to its great variability in quality and its potential for contamination [4]. Several physico-chemical parameters has been established as an indicator of water quality such as taste, color, odor, pH, electrical conductivity (EC), hardness, alkalinity, salinity, and presence of cations and anions [5]. High-quality drinking water has been defined as water that contains inclusions, which promote vitality and lack inclusions causing morbidity and mortality [5]. Because no water in nature is 100% pure, and hence different water sources will have varying degree of water inclusions, which directly or indirectly affect poultry performance and welfare. Many authors had studied the drinking water quality constituents and its effect on poultry performance [6-9].

Using of water with adequate physical, chemical, and microbiological quality is of a fundamental importance for poultry. Since many birds have access to the same water source, quality problems will affect a great number of birds. High levels of certain chemicals in water lead to changes in bird’s behavior and performance, via decrease body weight (BWT) and feed intake (FI) through prevention of nutrients absorption from feed ingredients. Items to be considered are total dissolved solids (TDS), pH, nitrate, sulfate, and salinity [10]. Studying the drinking water inclusions and their cumulative effects on growth performance was demonstrated by Zimmermann [5], who concluded that BWT was positively influenced by hardness, pH, and dissolved oxygen of drinking water and negatively influenced by its total aerobic bacterial number (TB). He added that feed conversion positively influenced by TB and negatively influenced by higher potassium (K) level in drinking water. Moreover, he said that mortality increased with nitrate and decreased with (K) level. In addition, water bacterial load has a serious impact on broiler flock. Grizzle *et al.*, [11] reported that broiler chickens exposed to drinking water contaminated with coliform bacteria (500 cfu/ml *Escherichia coli*) exhibited reduced BWT at 4 and 6 weeks of age. van der Sluis [12] reported that poor water quality may reduce the efficacy of vaccines and medications administered through water lines. There are extremely important interactions between drinking water contaminants and suboptimal nutritional status on performance and immune function of broiler chickens [13]. Field experience has conclusively shown that unobservable differences in water quality, from farm to farm and even from one well to another within a complex, can result in significant differences in bird performance, behavior, and welfare [5]. Moreover, as several elements can cause poor water quality, the interaction between elements is more significant in water quality problems than the simple fact of their presence [14]. There is an urgent need for updated methods and strategies for control of poor water issues and water borne diseases at the farm level. A number of treatment processes, such as filtration, coagulation, and solar radiation, are being used to modify the physiochemical properties of water [14]. Reverse osmosis (RO), is one of the most important water treatment technology that has become more and more applied in the treatment of water for livestock, horses, pigs, and poultry [15].

In Egypt, poultry production has gained great attention among wide sections of citizens, where most poultry farmers occupy the villages and peri-urban areas. Such areas usually lack in the continuous potable water supply. As a result, most households and poultry farmers have water storage tanks located on the rooftops of their homes or farms in order to have a constant supply of water, which challenged by several water quality issues. As water quality is unique within regions with limited studies concerning the quality of different water sources used as drinking water for poultry in Egypt. Consequently, the current study aimed at comparing different drinking water sources (tap, ground, stored and filtered water) which are the most commonly used in Egypt, on broiler chickens performance and welfare with exacting attention to carcass characteristics, hematological, biochemical effects, and immune response to newcastle disease virus (NDV) vaccine.

## Materials and Methods

The experiment was carried out for 6 weeks at the Research Poultry Farm Unit of Faculty of Veterinary Medicine Kafrelsheikh University, Egypt.

### Ethical approval

Animal Ethics Committee, Faculty of Veterinary Medicine, Kafrelsheikh University, Egypt, approved the protocol and conduct of the study.

### Experimental birds

A total of 204 unsexed day-old Indian river chickens purchased from a commercial hatchery at Kafr El-Sheikh city, Egypt were used in this study. The chickens were randomly allocated into four treatment groups of 51 birds each. Each treatment was divided into three replicates, 17 birds each. The treatments were distributed according to the source of water into, treatment one (T1); birds received farm tap water, treatment two (T2); birds received filtered tap water by using (RO mini 4) filter from water world Egypt (www.waterworld.egypt.com) First stage: Sediment filter, which remove sediments, insects, asbestos fiber and particles down to 5 μ. Second stage: Granular activated carbon filter that absorb heavy chlorine and chlorine by-products such as chloramines, trihalomethanes (THM) and tetrachloroethylene (TCE). Third stage: Carbon block filter, which remove additional chlorine and organic matter from water without the release of carbon fiber. Fourth stage: Which add essential minerals such as Ca, Mg and Na that adjust mineral substance to supplement water out of RO membrane. Treatment three (T3); birds received farm stored water (in stainless steel rooftop storage tank); Treatment four (T4), birds received underground water from a well of about 35 m depth at Kafr El-Sheikh city.

### Housing and management

The chickens in all groups (from day 0 to day 42) were brooded and raised on deep litter system and received the same standard management, hygienic, and environmental conditions. The stocking density was 11-12 bird/m^2^. Feed and water were provided ad libitum using manual plastic feeders and drinkers. Ration was formulated (Table-1) to meet the nutrient requirements for broilers [16]. Chickens were vaccinated against ND by oral administration in drinking water at days 7 and 18 using Hitchner and LaSota strains according to manufacturer recommendations respectively. The light program was set as 24 h continuous light during the first 3 days and 23 h light and 1 h dark (23L/1D) till the end of the experiment.

**Table-1:** Composition and nutrient composition of experimental diet.

Ingredient	Experimental diet (%)
Corn	55.5
Soybean meal	32.650
Sunflower oil	5.35
Fish meal	3.25
Dicalcium phosphate	1.5
Lime stone	1.0
L. Lysine	0.1
DL - Methionine	0.1
Salt	0.3
Premix	0.25
Nutrient composition	
Energy (Kcal/kg)	3200.71
Crude protein (%)	21.63

### Water sampling and analysis

Water samples for physico-chemical and bacteriological analysis were collected according to APHA, [17]. Physico-chemical analysis of water samples was carried out to determine color, odor, taste, temperature, pH, EC, total hardness, TDS, iron, nitrate, sulphate, phosphate, Na, Ca, Mg and Cl according to HACH, [18]. The total bacterial count of water samples was carried out according to the procedure described by Ayandirana *et al.*, [19]. Total coliform and fecal coliform count were enumerated by three-tube MPN procedure APHA, [17].

### Broiler performance

From day 0 to 42, Chickens were individually weighed weekly. Average live BWT and average live BWT gain (BWG) were calculated. FI and water intake (WI) were calculated daily for each treatment. Feed conversion ratio (FCR) and WI/FI ratio were calculated weekly for all treatments.

### Carcass and some internal organs weight

At day 42, 15 bird of an average BWT from each treatment were euthanized carefully and humanly (broilers were carefully euthanized via exsanguination from a neck cut that severed the carotid artery and jugular vein). The birds were kept for 5 min for bleeding and then dipped in a hot water bath for 2 min to facilitate the process of de-feathering. Manual evisceration was done to obtain carcass, gizzard, heart, liver, spleen and bursa weight. Absolute weight and relative weight of the carcass and internal organs to final BWT was calculated.

### Blood sampling and analysis

From each experimental treatment, 12 blood samples (5 ml each) were collected from a brachial vein at the day 28 of the experiment and from bleeding during euthanize at the day 42. From these two samples, 1 ml whole blood was taken in ethylenediaminetetraacetic acid tubes for making total and differential leucocytes count and estimation of heterophil/lymphocyte (H/L) ratio [20]. The other 4 ml blood was used for serum separation by centrifugation at 3000 rpm for 10 min. The serum samples were stored at −40°Ċ until used for assaying of the following parameters:

- Liver function indicators: Glutamic oxaloacetic transaminase (GOT) and glutamic pyruvic transaminase (GPT) enzymes [21], serum total proteins (TP), albumin (AL) and globulin (GL) [22]
- Kidney function indicators: Serum levels of uric acid [23]. Moreover, creatinine [24]
- Detection of antibody (Ab) titres against NDV by haemagglutination inhibition (HI) test: It was performed at the day 28 of the experiment following the second NDV vaccination with the LaSota strain at day 18 of broiler’s age [25].

### Statistical analysis

Data were tested for distribution normality and homogeneity of variance. Data was reported as mean ± standard error of the mean and analyzed by ANOVA using Minitab^®^ 15 (Minitab Inc., State College, PA, USA). The significance of difference among the different treatments was evaluated by Tukey’s test. The significance level was set at p>0.05.

## Results and Discussion

### Physico-chemical examination of water samples

This study was done to evaluate the impact of different water sources commonly used as drinking water in Egypt for poultry on performance, carcass responses, hematological, biochemical and immunological parameters that affect broiler health and welfare.

The results of this study showed that (Table-2) water quality parameters of different water sources (tap, RO treated water (RO), stored and well water) used in the current experiment were within the acceptable range for physico-chemical parameters of poultry water quality standards [26]. However, stored water at rooftop storage tanks recorded higher numerical values for pH, EC, TDS, alkalinity, salinity, potassium, chloride, sulfate, phosphate, total bacterial and coliform count among the examined water sources. Nevertheless, these increased values were still found within the acceptable range except for bacterial values. On the other hand, it recorded the lowest numerical values for hardness, Ca and Mg. These findings may emphasize the hypothesis of WHO [27], who stated that the storage of water in reservoirs creates favorable conditions for the self-purification of the stored water, in the same time it may cause undesirable changes in water quality. High values of the estimated parameters in stored water could be a consequence of undesirable conditions, which could be created by the production of algae. Also to pollution by birds and animals dropping and evaporation. The observed increase in pH in stored water could be a result of the activity of resident flora and or their death, which results in the release of an inorganic substance such as ammonia [28]. Moreover, these results may be attributed to the decay of chlorine residual in water with increase residence time of water in stored tanks. Where chlorine is in part being used to form disinfectant by-products (DBPs) where THMs and haloacetic acids are the two most significant, regulated DBPs of concern [29], which could increase the TDS and EC of water source.

**Table-2:** Physico-chemical and bacteriological examination of different water sources used in the experiment.

Parameters	Water sources				Poultry water quality* standard	
		
Physico-chemical examination	G1	G2	G3	G4	Level considered average	Maximum acceptable level
Color	Colorless	Colorless	Colorless	Colorless		
Odor	Inoffensive	Inoffensive	Inoffensive	Inoffensive		
Taste	Palatable	Palatable	Palatable	Palatable		
Temperature (°C)	20.5	20.4	20.4	20.5		
pH	8	8.04	8.2	8.2	6.5-7.8	5-8
EC (ms/cm)	471	463	1509	483	1000	
TDS (ppm)	236	232	755	242	0-1000	1000-3000
Hardness (μg/l)	120	150	75	100	0-75 soft 76-150 some what hard 151-300 hard >300 very hard	
Alkalinity (ppm as CaCo_3_)	157.4	147.5	619.7	147.5	100	300
Salinity (mg/l)	64	62	94	64		
Potassium (mg/l)	6.63	6.63	8.91	6.63	<300 no problem >300 satisfactory, depending on alkalinity and PH	
Calcium (mg/l)	36	36	20	32	60	
Magnesium (mg/l)	12.72	13.2	10.08	13.32	14	125
Sodium (mg/l)	30.4	31.3	31.3	32.4	50	150
Chloride (mg/l)	21.3	21.3	35.5	21.3	50	150
Iron (ppm)	<0.22	<0.22	<0.22	<0.22	0.2	0.3
Carbonate (mg/l)	0.00	0.00	36	0.0		
Bicarbonate (mg/l)	192	180	756	180		
Sulfate (mg/l)	26.4	31.9	40.8	46.08	15-40	200
phosphate	15.2	7.8	36.3	6.2		
Bacteriological examination						
Total bacterial count (cfu/ml)	170×10^3^	10.8×10^3^	210×10^3^	134×10^3^	0	1000
Total coli form count (cfu/ml)	2.8	0	251.4	500	0	50
Fecal coli form count (cfu/ml)	2	0	232.4	150.2	0	0

G1=Tap water, G2=Filter water, G3=Stored water, G4=Ground water,

* Adapted from Watkins (2008), EC=Electrical conductivity, TDS=Total dissolved solids

On the other hand, filtered water or RO treated water recorded lower numerical values for TDS, EC, alkalinity, sulfate and phosphate. It also showed a marked decrease in total bacterial count and absence of fecal coliform bacteria. Abdullah [1], reported similar findings. On the other hand, RO treated water recorded higher numerical values for hardness, Ca and Mg among the examined water sources. The low values of such estimated parameters could be attributed to the action of the three stages of water filter used in this experiment (as described later in materials and methods). In addition, RO membrane act as an ultrafiltration device, screening out particles, includes microorganisms that are physically too large to pass through the membrane’s pores. As well as, removal of compounds larger than 0.0001 μ and smaller than 0.1 μ in size [30]. However, such high values for minerals in RO water could be explained by the addition of essential minerals such as Ca, Mg and Na by the action of the post treatment (fourth) stage of used filter that adjust mineral substance to supplement water out of RO membrane.

Results of the bacteriological examination of the water sources illustrated in Table-2 showed that all water sources in all treatments except filtered water exceed the acceptable range of water quality standards of poultry for microbiological parameters [26]. The bacteriological examination recorded the worst results at (T3), especially fecal coliform which confirmed by a marked increase in phosphate concentration (36.3 mg/l) compared to the other water sources. The higher concentration of phosphate is considered as an indicator of fecal contamination [31,32]. The results that could be explained on the basis of the fact that water storage tanks provide increased residence time and aging of water [33,34]. As the water, aging have a negative impact on the quality of drinking water, including loss of chlorine residual [35] and re-growth of microorganisms.

The ability of water tanks to retain temperature is another factor adds to the bad quality of water in stored tanks. As remaining of water in direct sunlight leading to subsequent increasing in temperature which, influencing the growth rate of bacteria that have survived treatment processes [36]. Moreover, the design of household storage tanks and frequency of cleaning also appears to influence the microbial water quality of the stored water [32]. Finally, the difficulty in emptying the tanks completely allows the sediment building up that can act as a growth medium for microbes in the incoming water [33].

### Effect of water sources diversity on poultry performance

For judging the effect of drinking water quality characteristic that could affect directly or indirectly broiler performance, different live performance parameters were studied as an indicator of chicken welfare.

The performance parameters (BWT, BWG, FI, WI, WI/FI ratio, and FCR) per week showed no significant difference (p>0.05) among the experimental treatments of broiler chickens supplied with different water sources (Tables-3 and 4). The current result is consistent with several previous studies [6-9].

**Table-3:** Effect of water source diversity on BWT of broilers (g).

Treatments	1 day old	7 day old	14 day old	21 day old	28 day old	35 day old	42 day old
T1	38.08±0.7	108.2±2.4^a^	313.1±4.8^a^	628.6±29.0^a^	1322±16.9^a^	1685.2±72.1^ab^	2160±105
T2	38.25±0.6	119.1±3.8^b^	396.1±7.6^b^	786.0±20.4^b^	1230±8.4^b^	1807.9±46.7^a^	2262±100
T3	38.18±1.0	113.3±1.5^ab^	233±18^c^	644.2±15.4^a^	1236±23.3^b^	1613±41.7^ab^	2318±108
T4	38.13±1.1	117.2±0.9^ab^	359.8±13^ab^	699.2±13.7^a^	1223±5.8^b^	1486.7±55.7^a^	2082±87.0

Mean values in each column have not a common superscript are significant different at p<0.05, BWT=Body weight

**Table-4:** Effect of water source diversity on BWG/week (g) of broiler.

Treatments	1^st^ week	2^nd^ week	3^rd^ week	4^th^ week	5^th^ week	6^th^ week
T1	70.2±2.2^a^	204.8±4.4^a^	298.9±35.4^a^	693.4±30.8^a^	369.2±56.2^a^	475.1±74.4
T2	80.9±4.2^b^	276.9±5.8^b^	374±21.5^ab^	444±21.6^b^	578.9±35^b^	469.2±61.6
T3	75.2±2.1^ab^	119.7±19.1^c^	433±7.7^b^	591.8±30.8^c^	381.7±30.6^a^	667.8±73
T4	79.1±0.6^ab^	242.5±13.5^ab^	350.3±14.7^ab^	523.8±9.9^bc^	277.2±42.4^a^	567.7±32.8

Mean values in each column have not a common superscript are significant different at p<0.05, BWG=Body weight gain

The results in Table-4 showed that there was a significant difference (p<0.05) in the average BWG of broilers among the experimental treatments along the rearing period except at the 6^th^ week. BWG significantly reduced at 5^th^ week in T1, T3 and T4 groups which may be attributed to the sudden change that was occur in the temperature and humidity in this week which leads to such drop in BWG in this week which overcome by T2.

However, the results at Table-3 showed that birds received filtered water (T2) had the highest numerical values for most of the performance parameters as it recorded the highest average BWT at the day 7, 14, 21, 28, and 35.

On the other hand, (T2) group recorded a significant increase (p<0.05) in BWG in the 1^st^, 2^nd^, and 5^th^ week of the rearing period compared to the other experimental groups. Furthermore, (T2) group showed the lowest numerical values for WI and WI/FI ratio where these results may be account for low TDS and salinity of this source of water [4]. Ahmed [37] reported an increase in WI in the broiler with increased TDS in drinking water. As among the factors affecting WI is the amount and type of salts in water [38]. In this respect, Mushtaq *et al*. [39] suggested that the higher water consumption limits the gut capacity for FI and excretes nutrients in feces thus reduces weight. These findings could explain and support the current obtained results that achieved at (T2) group, which showed that, with low TDS and salinity of water source, WI/FI ratio decrease with subsequent increase live BWT and BWG. This suggestion was fully agreed with earlier findings of Ahmed [37], who reported a negative correlation between feed efficiency and BWG with an increase in drinking water TDS.

Moreover, T2 group showed higher poultry growth and improved FCR which could be attributed initially to the high magnesium content of this source of water [4], then to the reduction of total aerobic bacteria and *E. coli* respectively [5,40]. Where all the above-mentioned findings of improved performance indices for (T2) group may emphasize the hypothesis of Blake and Hess [41], which approved that high level of bacterial contaminants, minerals, or other pollutants in drinking water can have detrimental effects on normal physiological properties resulting in inferior performance. Moreover, these results may imply that with a low bacterial load in the offered drinking water source it could achieve decrease pressure on the animal immune system. Thus more nutrients will be available for productive functions such as growth.

From the results illustrated at Table-5, it is clearly noticeable that there was a similar trend of improving FCR observed at (T3) were recorded as the highest weight gain and the lowest FCR compared to the other experimental treatments. Although of such improved trend of FCR at (T3) group, but (T2) group recorded lower FI which may be considered economically better. Such improved BWG and FCR at (T3) group could be attributed to high concentration of potassium and chloride ions in such water source comparing to the other water sources. Potassium chloride (KCl) supplementation in drinking water has been reported to increase the BWG and survivability in chickens to varying degrees [42]. On the same line, Zimmermann *et al*. [43] and Abbas *et al*. [4], reported a positive correlation between FCR and sulfate level at water source, which achieved at the stored water source.

**Table-5:** The effect of water source diversity on total BWG, FI/WI ratio and FCR.

Treatments	BWG (g) for bird/week	FI (g) for bird/week	WI (ml) for bird/week	FI/WI ratio	FCR
T1	351.9±88.7	697±173	252.4±56.6	2.81±0.38	2.01±0.03
T2	370.6±71.0	730±146	228.8±51	3.34±0.30	1.97±0.04
T3	378.2±98.5	742±204	249.2±58.5	2.89±0.29	1.95±0.06
T4	340.1±74.7	732±168	236.9±52.5	3.19±0.40	2.12±0.05

BWG=Body weight gain, FI=Feed intake, WI=Water intake, FCR=Feed conversion ratio

### Effect of water sources diversity on carcass characteristic

Studying the carcass responses in this experiment (Table-6 and 7) revealed that, there was no significant difference (p>0.05) in the relative weights (as a percent of final BWT) of eviscerated carcass and internal organs (liver, spleen and heart), for exception bursa and gizzard among the experimental treatments. The results that are in full agreement with authors [7-9], who reported that the different water sources had no significant effect on carcass characteristics. Birds at (T3) showed the higher eviscerated carcass percentage compared with the other experimental treatments. These may be attributed to high numerical values of TDS (755 ppm) and salinity (94 mg/l) in this source of water, which lead to increase WI and subsequent increase water accumulation in muscles and tissues [39]. The increase in weight and size of most of the body organs is an indication of stress condition.

**Table-6:** Effect of water source diversity on carcass characteristics and internal organ weight of broilers.

Treatments	Eviscerated carcass (g)	Eviscerated carcass (%)	Liver (g)	Spleen (g)	Bursa (g)	Heart (g)	Gizzard (g)
T1	1555.3±65.4	72	48.61±3.72	2.0±0.11	2.44±0.21^a^	9.53±0.48^a^	33.55±1.37^a^
T2	1678±77	74.18	47.81±2.14	2.161±0.15	2.85±0.25^ab^	10.87±0.42^ab^	34.11±1.33^a^
T3	1727±87.2	74.50	52.31±3.05	2.38±0.13	3.54±0.35^b^	11.93±0.58^b^	38.54±1.18^ab^
T4	1541.7±64.5	74.05	47.81±2.86	2.26±0.24	2.18±0.16^a^	9.59±0.46^a^	40.16±1.73^b^

Mean values in each column have not a common superscript are significant different at p<0.05

**Table-7:** Effect of water source diversity on carcass characteristics, weight (g) and relative weights of internal organs to final body weight (%) of broilers.

Treatments	Eviscerated carcass		Liver		Spleen		Bursa		Heart		Gizzard	
					
	(g)	(%)	(g)	(%)	(g)	(%)	(g)	(%)	(g)	(%)	(g)	(%)
T1	1555.3±65.4	73.89±4.00	48.61±3.72	2.275±0.157	2.0±0.1	0.1±0.1	2.44±0.1^a^	0.12±0.1^ab^	9.53±0.48^a^	0.45±0.2	33.55±1.7^a^	1.60±0.9^ab^
T2	1678±77	75.24±3.44	47.81±2.14	2.1302±0.071	2.16±0.5	0.1±0.1	2.85±0.5^ab^	0.13±0.1^ab^	10.87±0.4^ab^	0.49±0.2	34.11±1.3^a^	1.53±0.07^a^
T3	1727±87.2	76.96±5.87	52.31±3.05	2.350±0.219	2.38±0.3	0.11±0.1	3.54±0.35^b^	0.16±0.2^b^	11.93±0.58^b^	0.53±0.4	38.54±1.8^ab^	1.71±0.10^ab^
T4	1541.7±64.5	75.01±3.26	47.81±2.86	2.2978±0.091	2.26±0.4	0.11±0.1	2.18±0.16^a^	0.10±0.1^a^	9.59±0.46^a^	0.47±0.3	40.16±1.7^b^	1.98±0.12^b^

Mean values in each column have not a common superscript are significant different at p<0.05

In this study, T2 showed lower liver, spleen and gizzard relative weights than other treatments. These results may be attributed to low TDS and salinity of such water sources [1,44]. On the same concept, these findings also could explain the results of a significant increase in bursa relative weight at T2 that reflect the wellbeing of the bird condition, growth performance, and immune function. The assessing bursa weight and the bursa/BWT ratio gave the most consistent and reliable indication of stress [44].

Currently, the results imply that broiler chickens drink filtered water had the better immune response and disease resistance. In this respect, Katanbaf *et al*. [45] reported that the increase in the relative organs weight is considered as an indication of the immunological advances. This established an increase in bursa weight in (T2) group might be reinforced by the effect of RO treatment to produce pure water with low chemical contaminants. The findings that is in agreement with Vodela *et al*. [13], who observed atrophy of the bursa of Fabricius in all the birds exposed to chemical mixture in drinking water which exaggerated by vitamin and mineral diet deficiency. In respect to the low bacterial count obtained at RO water, it could add another explanation to increased bursal weight in T2 group. Atef *et al*. [46] reported a decrease in bursa weights as a result of high nitrate or pathogenic bacterial challenge.

### Effect of water sources diversity on total leukocytes count (TLC) and H/L ratio

TLC is considered one of the most important parameter that is used for assessing physiological stress in birds. Moreover, the H/L ratio is considered one of the most important indicators used for evaluation of stress response and immune status [47]. Data presented at Table-8 showed that, there was a significant difference (p<0.05) in TLC, heterophil (H%), lymphocyte (L%) and H/L ratio among the experimental treatments on the day 28 and 42. From these results, it was found that T2 was represented by the lower values of TLC, H%, and H/L ratios and the higher values of L% compared to the other treatments on the days 28 and 42. These results could be referred to an adequate and healthy nutrition, as well as reflect the degree of wellbeing status of chickens received filtered water with low salinity and TDS in which all stress markers has been recorded at low levels. The results were in agreement with Ahmed [37], who reported an increase in H/L ratio in chickens which given saline water. Moreover, filtered water recorded lowest TBC and fecal coliform count than other water sources. These data could emphasize the findings has been previously approved by Grizzle *et al*. [11], who declared that administration of drinking water containing coliform bacteria (*E. coli* “500 cfu/ml”) could induce inflammatory response and compromise the immune response of animal. Where heterophils are the primary phagocytic leukocyte, and proliferate in circulation in response to infections, inflammation, and stress [48].

**Table-8:** Effect of water source diversity on hematological parameters of broilers.

Day	T1	T2	T3	T4
Day 28				
TLC/cu mm	40767±894^a^	29233±1422^b^	34633±665^c^	38233±393^ac^
Heterophils (H) %	5.17±0.48^a^	3.17±0.31^b^	3.67±0.42^ab^	5.33±0.56^a^
Lymphocytes (L) %	82±1.03^a^	87.67±0.49^b^	85.67±0.56^b^	82±0.82^a^
H/L ratio	0.063±0.007^a^	0.036±0.004^b^	0.043±0.005^ab^	0.065±0.007^a^
Day 42				
TLC/cu mm	36747±853^a^	22892±848^b^	38890±833^c^	31958±998^a^
Heterophils (H) %	4.6±0.31^a^	3.08±0.37^b^	5.55±0.3^a^	3.92±0.26^ab^
Lymphocytes (L) %	78.67±4.41^a^	89.67±0.90^b^	82.22±0.71^ab^	86.25±0.68^ab^
H/L ratio	0.073±0.019^a^	0.035±0.004^b^	0.068±0.004^ab^	0.046±0.003^ab^

Mean values in each column have not a common superscript are significant different at p<0.05, TLC=Total leukocytes count

### Effect of diversity of water sources on liver, kidney tests and antibody titer against NDV vaccine

Liver and kidney function indicators were estimated and used as physiological markers for assessing the health status and welfare of broilers. Concerning the impact of different water sources on liver indicators, current findings illustrated at Table-9 revealed that, (T2) had a significant (p<0.05) decrease in serum GOT and GPT (except for GOT at day 42), with these changes being more evident at younger ages (day 28). Where serum GOT and GPT enzymes were used as a biomarker of liver function and liver injury as their increase activity usually related to degeneration and liver damage [49]. The results of lower liver enzymes activity were correlated with the previous findings of lower liver weight of (T2) group supporting the suggestion of better wellbeing and health status of such group. Where stress condition on chicken could elevate the activity of both GOT and GPT as a consequence of higher corticosteroid level [50]. Based on the present results, the decreased liver enzymes activity in (T2) was reinforced by the effect of RO treatment device in offering pure water with low organic matter and chemical contaminants such as TCE, which has been showed to induce experimentally liver tumors in mice [51]. Moreover, data presented in Table-9 pointed out a significant (p<0.05) increase in AL and GL fractions (except AL fraction at day 42) in birds received RO water (T2) compared with the other groups, which accompanied with a significant (p<0.05) increase in TP and decrease in A/G ratio at day 28 and 42. The findings that disagreed with those results that reported a significant increase (p<0.05) in TP, AL and GL levels in pigs [52], and poultry [1,37] given minerals water. As well as, the decrease in A/G ratio were in contrary to those results given by Patience *et al*. [53], who reported that pigs receiving RO treated water showed high A/G ratio. This contrary result may be attributed to different water quality characteristics and species differences. In the present study, hyperproteinemia and hyperglobulinemia has been obtained in (T2) group, could be a possible explanation to the humoral immune response observed by a significant increase (p<0.05) in total antibody titer against NDV in T2 group at the day 28. As GL level has been used as an indicator of immune responses and act as a source of antibody production. Griminger [54] stated that high GL level and low A/G ratio were considered better indicators for disease resistance and immune response. At the same time, these findings were in correlation with increased lymphoid organs weight in T2. The results that is in agreement with Ahmed [37] who demonstrated a significant increase at NDV antibody titer in chickens received drinking water with low TDS levels (265 and 2610 ppm). These results may imply that broiler chickens administered filtered water had better immunity. Where the established enhancement in immune response associated with drinking filtered water in this study may be account for low bacterial load in filtered water, where their presence could reduce the effectiveness of vaccination [55]. As well as, these good results of immune response may be achieved by the ability of RO treatment to reduce the concentration of dissolved solids, including a variety of ions and metals and very fine suspended particles such as asbestos that may be found in water. The findings that is in harmony with those results given by Vodela *et al*. [13], who demonstrated a significant linear decrease in the levels of antibodies in chicken sera that cross-reacted with rabbit red blood cells associated with increasing concentrations of chemicals in drinking water. In addition, this enhancement effect of RO water to bird immunity could be the results of high Ca and Mg in this water source. The supposition that may emphasize the hypothesis of Carpentieri *et al*. [56], who declared that Ca and Mg were necessary for the growth of B-cells and T-cells of humans. Continuously, data of uric acid, which is the major end product of protein metabolism in poultry [57] revealed that drinking filtered water significantly (p<0.05) reduced serum uric acid (urea) and creatinine concentration (except uric acid at day 42) compared with the other experimental treatments. This decrease was more marked at younger age. The results that is in agree with Abdullah [1], who reported an increase in serum uric acid in poultry received high saline water, in which contribute increase of fowl serum urea as a result of diffusion from kidney due to kidney failure [58]. From another point of view, these results could referee to the better utilization of protein and amino acid digestibility. It could be interpreted by low WI/FI ratio results obtained for T2 group which subsequent decrease the gastric emptying rate that considered the possible mechanism to improve the protein digestion in the gastrointestinal tract [59].

**Table-9:** Effect of water source diversity on liver and kidney function indicators and antibody titers against NDV in broilers.

Examined parameters	T1	T2	T3	T4
Day 28				
Liver function indicators				
GOT	239.7±11^b^	178.8±6.05^a^	233.8±10.9^b^	235.3±11.1^b^
GPT	5.38±0.21^c^	3.38±0.26^a^	4.80±0.10^bc^	4.38±0.28^b^
TP	3.10±0.09^a^	3.56±0.07^b^	2.97±0.05^a^	3.23±0.08^a^
AL	0.99±0.09^ab^	1.1±0.04^a^	0.87±0.04^b^	0.87±0.04^b^
GL	2.0±0.05^a^	2.56±0.05^b^	2.1±0.04^a^	2.37±0.06^c^
AL/GL ratio	0.56±0.06^a^	0.39±0.01^b^	0.42±0.0224^b^	0.37±0.02^b^
Kidney function indicators				
Urea	4.88±0.27^a^	2.89±0.23^c^	4.03±0.21^b^	4.02±0.20^b^
Creatinine	0.35±0.01^a^	0.22±0.01^c^	0.29±0.004^b^	0.26±0.01^b^
Antibody titres against NDV	6.05±0.29^a^	8.86±0.23^b^	8.48±0.25^b^	8.26±0.19^b^
Day 42				
Liver function indicators				
GOT	192.42±7.27	189.4±10.2	192±6.67	196.75±8.56
GPT	4.98±0.16^b^	4.14±0.18^a^	5.13±0.11^b^	4.86±0.17^b^
TP	2.93±0.06^a^	3.41±0.09^b^	3.08±0.07^a^	2.92±0.06^a^
AL	1.01±0.04	0.87±0.05	0.93±0.07	0.87±0.05
GL	1.93±0.04^a^	2.54±0.06^b^	2.15±0.04^c^	2.05±0.06^ac^
AL/GL ratio	0.52±0.02^b^	0.34±0.02^a^	0.43±0.04^ab^	0.43±0.03^ab^
Kidney function indicators				
Urea	4.58±0.32	3.56±0.28	4.23±0.18	4.57±0.40
Creatinine	0.37±0.01^a^	0.23±0.01^b^	0.32±0.01^c^	0.31±0.01^c^

Mean values in each column have not a common superscript are significant different at p<0.05, NDV=Newcastle disease virus, GOT: Glutamic oxaloacetic transaminase, GPT=Glutamic pyruvic transaminase, TP=Total proteins, AL=Albumin, GL=Globulin

## Conclusion

This study revealed that the broiler chickens could tolerate and grow well under using different water sources, as long as the water constituents present within the acceptable range of drinking water quality standards. However, RO treated water propose an improved health condition and welfare which draw an attention to the importance of improving water sources supplied for poultry in term of water treatment. From economic point of view, stored water received group may be represented as a higher performance group, but actually RO water received group may be equally or may be much better than stored water received group as they recorded lower FI, which considered economically better. Therefore, more attention should be paid to stored water quality at farm reservoir through good maintenance and frequent cleaning of water tanks with continuous sampling in order to monitor its quality.

## Authors’ Contributions

NE designed the study. NE, RAM and FA carried out the experiment. RAM analyzed the samples and, drafted the final manuscript. NE and FA reviewed and revised the manuscript. All authors read and approved the final manuscript.

## References

[1] Abdullah A.M (2011). Impact of different locations water quality in Basra province on the performance and physiological changes in broiler chickens. Pak. J Nutr.

[2] Jafari R.A, Fazlara A, Govahi M (2006). An investigation into Salmonella and faecal coliform contamination of drinking water in broiler farms in Iran. Int. J. Poult. Sci.

[3] Kellems R.O, Church D.C (2002). Livestock Feeds and Feeding.

[4] Abbas T.E, Elfadil A.E, Omer H.A (2008). Drinking water quality and its effects on broiler chickens performance during winter season. Int. J. Poult. Sci.

[5] Zimmermann N.G, Douglass L (1998). A survey of drinking water quality and its effects on broiler growth performance on Delmerva. Poult. Sci.

[6] Abbas T.E, ELzubeir E.A, Arabbi O.H, Mohamed H.E (2010). Drinking water quality I:Effects on broiler chickens performance during summer season. Res. J. Anim. Vet. Sci.

[7] Folorunsho O.R, Laseinde E.A, Onibi G.E (2012). Performance, hematology and carcass characteristics of broiler chickens given water from different sources. Niger. J. Anim. Prod.

[8] Asaniyan E.K, Adene I.C (2013). Influence of drinking water sources on haematological indices of broiler chicken Proceeding 38 Conference of Nigerian Society for Animal Production.

[9] Ibitoye E.B, Dabai Y.U, Mudi L (2013). Evaluation of different drinking water sources in Sokoto North-West Nigeria on performance, carcass traits and haematology of broiler chickens. Vet. World.

[10] Reutor R (2010). Water is the most important nutrients Nobel foundation. Agricultural Division.

[11] Grizzle J.M, Armbrust T.A, Bryan M.A, Saxton A.M (1997). The effect of water nitrate and bacteria on broiler growth performance. J. Appl. Poult. Res.

[12] Van der Sluis W (2002). Water quality is important but often over estimated. World Poultry.

[13] Vodela J.K, Lenz S.D, Renden J.A, McElhenney W.H, Kemppainen B.W (1997). Drinking water contaminants (arsenic, cadmium, lead, benzene, and trichloroethylene). 1. Interaction of contaminants with nutritional status on general performance and immune function in broiler chickens. Poult. Sci.

[14] Li L (2009). Clean drinking water is crucial in enhancing animal productivity. 17th Annual ASAIM SEA Feed Technology and Nutrition Workshop.

[15] Olkowski A.A (2009). Livestock Water Quality:A Field Guide for Cattle, Horses, Poultry and Swine.

[16] NRC (1994). Nutrient Requirements of Poultry.

[17] APHA (2005). Standard Methods for Examination of Water and Waste Water.

[18] HACH Company (2003). Hach Water Analysis Handbook.

[19] Ayandirana T.A, Ayandelea A.A, Dahunsib S.O, Ajalaa O.O (2014). Microbial assessment and prevalence of antibiotic resistance in polluted Oluwa River Nigeria. Egypt. J. Aquat. Res.

[20] Dein F.J (1984). Laboratory manual of avian hematology.

[21] Reitman S, Frankel S (1957). A colorimetric method for the determination of serum glutamic oxaloacetate and glutamic pyruvic transaminase. Am. J. Clin. Pathol.

[22] Armstrong W.D, Carr C.W (1964). Physiological Chemistry Laboratory Directions.

[23] Trinder P (1969). Enzymatic colorimetric method for determination uric acid. Ann. Clin. Biochem.

[24] Hare R.S (1950). Endogenous creatinine in serum and urine. Proc. Soc. Exp. Biol. Med.

[25] Majiyagbe K.A, Hitchner S.B (1977). Antibody response to strain combinations of Newcastle disease virus as measured by haemagglutination inhibition. Avian Dis.

[26] Watkins S (2008). Water Identifying and correcting challenges. Avian Advice.

[27] WHO (1996). Guidelines for Drinking-Water Quality:Health Criteria and Other Supporting information.

[28] Eniola K.I, Obafemi D.Y, Awe S.F, Yusuf I.I, Falaiye O.A, Olowe A.O (2007). Effects of containers and storage conditions on bacteriological quality of borehole water. Nat. Sci.

[29] Al-Jasser A (2007). Chlorine decay in drinking-water transmission and distribution systems:Pipe service age effect. Water Res.

[30] Koyuncu I, Arikan O.A, Wiesner M.R, Rice C (2008). Removal of hormones and antibiotics by nanofiltration membranes. J. Membr. Sci.

[31] Carter T.A (1985). Drinking water quality for poultry. Poult. Dig.

[32] Cynthia A.S (2010). Impact of Tank Material on Water Quality in Household Water Storage Systems in Cochabamba, Bolivia. A Master’s Thesis Department of Civil & Environmental Engineering College of Engineering. University of South Florida.

[33] Tokajian S, Hashwa F (2003). Water quality problems associated with intermittent water supply. Water Sci. Technol.

[34] Tokajian S, Hashwa F (2004). Microbial quality and genotypic speciation of heterotrophic bacteria isolated from potable water stored in household tanks. Water Qual. Res. J. Can.

[35] Robert M.C, Abdesaken F, Boulos P.F, Mau R.E (1996). Mixing in distribution system storage tanks:Its effect on water quality. J. Environ. Eng.

[36] Donlan R.M, Pipes W.O, Yohe T.L (1994). Biofilm formation on cast iron substrata in water distribution systems. Water Res.

[37] Ahmed A.S (2012). Performance and immune response of broiler chickens as affected by different levels of total dissolved solids in drinking water under hot arid environments. Anim. Prod. Sci.

[38] Mamabolo M.C, Casey N.H, Meyer J.A (2009). Effects of total dissolved solids on the accumulation of Br, AS and PB from drinking water in tissues of selected organs in broilers. S. Afr. J. Anim. Sci.

[39] Mushtaq M.M, Pasha T.N, Saima A.M, Mushtaq T, Parvin R, Farooq U, Mehmood S, Iqbal K.J, Hwangbo J (2013). Growth performance, carcass traits and serum mineral chemistry as affected by dietary sodium and sodium salts fed to broiler chickens reared under phase feeding system. Asian Australas J Anim. Sci.

[40] Samanta S, Halder S, Ghosh T.K (2010). Comparative efficacy of an organic acid blend and bactiracin methylene disalicylate as growth promoters in broiler chickens effect on performance, gut histology and small intestinal milieu. Vet. Med. Int.

[41] Blake J.B, Hess J.P (2001). Evaluating water quality for poultry.

[42] Ahmad T, Khalid T, Mushtaq T, Mirza M.A, Nadeem A, Babar M.E, Ahmad G (2008). Effect of potassium chloride supplementation in drinking water on broiler performance under heat stress conditions. Poult. Sci.

[43] Zimmermann N.G, Dhillon A.S, Barton T.L, Andrews L.D (1993). Relationship of drinking water quality and broiler performance in Washington State. Poult. Sci.

[44] Vahdatpour T.K, Nazer Adl Y.E, Nezhad N.M, Sis S.R, Vahdatpour S (2009). Effects of corticosterone intake as stress-alternative hormone on broiler chickens:Performance and blood parameters. Asian J. Anim. Vet. Adv.

[45] Katanbaf M.N, Dunnington E.A, Siegel P.B (1989). Restricted feeding in early and late-feathering chickens. Growth and physiological responses. Poult. Sci.

[46] Atef M, Abo-Norage M.A, Hanafy M.S, Agag K.E (1991). Pharmacotoxicological aspects of nitrate and nitrite in domestic fowls. Br. Poult. Sci.

[47] Mohamed R.A, Eltholth M.M, El-Saidy N.R (2014). Rearing broiler chickens under monochromatic blue light improve performance and reduce fear and stress during pre-slaughter handling and transportation. Biotechnol. Anim. Husbandry.

[48] Thrall M.A (2004). Hematology of amphibians. Veterinary Hematology and Clinical Chemistry:Text and Clinical Case Presentations.

[49] Lumeij J.T, Kanedo J.J, Harvey J.W, Bruss M.L (1997). Avian clinical biochemistry. Clinical Biochemistry of Domestic Animal.

[50] Siegel H.S (1995). Stress, strains and resistance. Br. Poult. Sci.

[51] Guyton K.Z, Hogan K.A, Scott C.S, Cooper G.S, Bale A.S, Kopylev L, Barone S, Makris S.L, Glenn B, Subramaniam R.P, Gwinn M.R, Dzubow R.C, Chiu W.A (2014). Human health effects of tetrachloroethylene:Key findings and scientific issues. Environ. Health Perspect.

[52] Maenz D.D, Patience J.F, Wolynetz M.S (1994). The influence of the mineral level in drinking water and thermal environment on the performance and intestine fluid flux of newly weaned pigs. J. Anim. Sci.

[53] Patience J.F, Beaulieu A.D, Gillis D.A (2004). The impact of ground water high in sulfate on growth performance, nutrient utilization and tissue mineral levels of pigs housed under commercial conditions. J. Swine Health Prod.

[54] Griminger P, Sturkie P.D (1986). Avian Physiology. Lipid metabolism.

[55] Florencio M, Tosar A, Sactidrian S (1990). Effect of tannic acid on the immune response of growing chickens. Anim. Sci. J.

[56] Carpentieri U, Myers J, Daeschner C.W, Haggard M.E (1988). Effects of iron, copper, zinc, calcium and magnesium on human lymphocytes in culture. Biol. Trace. Elem. Res.

[57] Sturkie P.D (1986). Avian Physiology.

[58] Harr K.E (2002). Clinical chemistry of companion avian species. A review. Vet. Clin. Pathol.

[59] Kommera S.K, Mateo R.D, Neher F.J, Kim S.W (2006). Phytobiotics and organic acids as potential alternatives to the use of antibiotics in nursery pig diets. Asian Australas J. Anim. Sci.

